# Short-term therapeutic effects of low-dose cytarabine plus surgical resection on elderly patients with trigeminal nerve tumor and safety observation

**DOI:** 10.12669/pjms.311.6440

**Published:** 2015

**Authors:** Xiang-Sheng Li, Wei-Long Yang, Fa-Zheng Shen, Guo-Jun Gao, Ji-Wei Ma, Bao-Zhe Jin

**Affiliations:** 1 Xiang-Sheng Li, Department of Neurosurgery, The First Affiliated Hospital of Xinxiang Medical University, Weihui 453100, PR China.; 2 Wei-Long Yang, Department of Neurosurgery, The Third Affiliated Hospital of Xinxiang Medical University, Xinxiang 453003, PR China.; 3 Fa-Zheng Shen, Department of Neurosurgery, The First Affiliated Hospital of Xinxiang Medical University, Weihui 453100, PR China.; 4 Guo-Jun Gao, Department of Neurosurgery, The First Affiliated Hospital of Xinxiang Medical University, Weihui 453100, PR China.; 5 Ji-Wei Ma, Department of Neurosurgery, The First Affiliated Hospital of Xinxiang Medical University, Weihui 453100, PR China.; 6 Bao-Zhe Jin, Department of Neurosurgery, The First Affiliated Hospital of Xinxiang Medical University, Weihui 453100, PR China.

**Keywords:** Cytarabine, Dose, Surgical resection, Trigeminal nerve tumor, Safety

## Abstract

**Objective:**

To evaluate the short-term therapeutic effects of low-dose cytarabine plus surgical resection on elderly patients with trigeminal nerve tumor and to observe the safety.

**Methods:**

A total of 120 elderly patients with trigeminal nerve tumor were divided into a treatment group and a control group by random draw (n=60), and both groups were subjected to resection by stereotactic image-guided endoscopic nasal surgery. Afterwards, the control group was administered with high-dose cytarabine while the treatment group was given low-dose cytarabine for 14 days.

**Results:**

Both groups completed treatment, but the effective rate of the treatment group (96.7%) was significantly higher than that of the control group (83.3%) (P < 0.05). The pain scores of the two groups were similar at T0, T1 and T2, but the score of the treatment group at T2 was significantly different from those at T0 and T1 (P < 0.05). During treatment, the treatment group was significantly less prone to complications such as headache, vomiting, vision impairment, nausea and local swelling than the control group (P < 0.05). During three months of follow-up, the appetite, sleep and daily living scores were significantly higher than those of the control group (P < 0.05).

**Conclusion:**

Stereotactic image-guided surgery was able to treat trigeminal nerve tumor well, and the effect was enhanced by low-dose cytarabine that improved postoperative outcomes and quality of life by dramatically decreasing complications.

## INTRODCUTION

Trigeminal nerve tumor, which only accounts for about 0.2%-1% of all intracranial tumors,^1^ originates from semilunar ganglion of the trigeminal nerve and grows slowly outside the dura mater of the middle cranial fossa. It severely affects patients' quality of life by invading adjacent cranial nerves, leading to severe, paroxysmal pain.^2^ The pathogenesis of trigeminal nerve tumor remains unclear, which has been mostly ascribed to microvascular compression of the trigeminal nerve root. However, the paroxysmal pain cannot be explained.^3^^,^^4^ Besides, trigeminal nerve tumor-induced pain has been related with the impaired inhibitory function of spinal trigeminal nuclei.^5^

It is now well-established that intervention on pathological nerve conduction exerts satisfactory effects by blocking or mitigating pain,^6^ which is mainly realized by radiofrequency ablation, endoscopic surgery, craniotomy for microvascular decompression, peripheral trigeminal nerve avulsion, trigeminal sensory rhizotomy and micro-balloon compression, etc. Particularly, endoscopic approach to the pterygopalatine fossa via the nasal cavity can be conducted directly and minimally invasively, which needs a marker for the anatomical position. To this end, CT and stereotactic image guidance are helpful and thus can remarkably reduce complications owing to accurate surgical procedure and targeted therapeutic effects.^7^

In addition, trigeminal nerve tumor-induced pain among elderly patients may be associated with the pathological and physiological changes of peripheral and central nervous systems, such as abnormal impulse of afferent nociceptive neurons. Therefore, additional drug therapy is required.^8^ On the other hand, cytarabine, as a pyrimidine antimetabolite that mainly targets the S phase of cell cycle, interferes with the proliferation of cells mainly through inhibiting cellular DNA synthesis, thus benefiting the treatment of trigeminal nerve tumor.^9^ High dose for cytarabine means 1.0-3.0 g/m^2^ body surface area. However, the adverse reactions of this drug increase with rising dose, so low dose (10 mg/m^2^) is usually recommended, at which it binds DNA and then kills tumor cells.^10^

In this study, we aimed to evaluate the short-term therapeutic effects of low-dose cytarabine plus surgical resection on elderly patients with trigeminal nerve tumor and to observe the safety.

## METHODS

***Clinical baseline data: ***A total of 120 elderly patients with trigeminal nerve tumor treated in our hospital from August 2010 to November 2013 were selected in this study.

***Inclusion criteria:*** Diagnosed as trigeminal nerve tumor by history of diseases, physical signs and radiographical data; ≥60 years old; with symptoms such as masticatory muscle weakness, diplopia, facial hypoesthesia and corneal reflex decrease; painful in the dominated area of the maxillary nerve; with written consent.

***Exclusion criteria:*** Intolerant or unwilling to accept surgeries; unable to complete surgeries due to mental disorders; with abnormal liver, kidney functions and blood clotting; with secondary trigeminal neuralgia; with psychological and mental disorders that could not be treated by drugs or psychotherapy.

The eligible patients were divided into a treatment group and a control group by random draw (n=60), and the two groups had similar gender ratio, age, body mass index, case distribution and disease course (P > 0.05) (Table-I).

***Treatment methods: ***All patients were first subjected to resection by endoscopic nasal surgery after 8 hour of food deprivation and 4 hour of water deprivation. Spiral CT thin-section axial scanning was performed one day before surgery from the supraorbital ridge to the angle of the mandible. The foramen ovale and surrounding important structures were observed and marked, and the angle and distance between the foramen ovale and the sphenopalatine foramen were estimated to simulate surgical positioning. During surgery, the patient was subjected to general anesthesia by tracheal intubation in the supine position on a routinely sterilized surgical drape. Then the nasal cavity was examined, and the nasal mucous membrane was sterilized, incised with an electric knife at the posterior edge of the membranous fontanelle of the maxillary sinus until the sphenopalatine foramen, while keeping the maxillary sinus mucosa intact. A part of posterior wall of the maxillary sinus or substance of the posterior ethmoid bone was removed by a rongeur to enter the pterygopalatine fossa. Under the guidance of CT, the periosteum of the pterygopalatine fossa was incised to seek maxillary nerves and the foramen ovale, and nerves adjoining to the foramen ovale were cut off. The patients feeling painful in the branches of the trigeminal nerve were subjected to electronic cutting of the supraorbital nerve. After surgeries, all patients were intravenously infused with antibiotics, and their noses were packed with hemostatic materials.

***Drug administration: ***Control group: After surgeries, this group was intravenously infused with high-dose (1.0-3.0 g/m^2^ body surface area) cytarabine for 14 consecutive days (bid). Treatment group: After surgeries, this group was intravenously infused with low-dose (10 mg/m^2^ body surface area) cytarabine for 14 consecutive days (bid).

***Observation indices: ***Standards for therapeutic effects: Markedly effective: Main clinical symptoms basically disappeared, and pain was moderately relieved (by half and above). Improved: Main clinical symptoms were partly alleviated, and pain was slightly relieved (by 1/4-1/2). Ineffective: Neither main clinical symptoms nor pains were relieved or even aggravated.

***Pain grading:*** Pain was graded before (T0) and 1 h (T1) and 14 d (T2) after surgeries by using visual analogue scale, with 0 being painless and 10 being extremely painful.

***Safety observation:*** The complications of patients, including headache, vomiting, vision impairment, nausea and local swelling, were observed during treatment.

***Investigation on quality of life:*** All patients were followed up for 3 months and investigated in the dimensions of appetite, sleep, daily living, social life and interest in life. A higher score meant better quality of life.

***Statistical analysis: ***All data were analyzed by SPSS19.0. The categorical data were expressed as mean ± standard deviation (x ± s), and inter-group and intra-group comparisons were performed by t test and repeated measures analysis of variance respectively. The numerical data were compared by Chi-square test. P < 0.05 was considered statistically significant.

## RESULTS

***Therapeutic effects: *** Both groups completed treatment, but the effective rate of the treatment group (96.7%) was significantly higher than that of the control group (83.3%) (P < 0.05) (Table-II).

***Pain scores: ***The pain scores of the two groups were similar at T0, T1 and T2, but the score of the treatment group at T2 was significantly different from those at T0 and T1 (P < 0.05) (Table-III).

***Complications: ***During treatment, the treatment group was significantly less prone to complications such as headache, vomiting, vision impairment, nausea and local swelling than the control group (P < 0.05) (Table-IV).

***Quality of life: ***During three months of follow-up, the appetite, sleep and daily living scores were significantly higher than those of the control group (P < 0.05) (Table-V).

## DISCUSSION

Trigeminal nerve tumor rarely occurs and cannot be found early until it grows to trigger trigeminal neuralgia. Firstly, the posterior sensory root of the trigeminal nerve was involved and damaged, or the facial nerve was compressed. With enlarging tumor, the auditory nerve, the facial nerve and the trigeminal nerve are injured, which are accompanied by cerebellar damage and compression-induced brainstem shift. Of all the symptoms, trigeminal neuralgia is most common^11^ that is clinically manifested as intermittent, intolerant, extreme pain, involving at least two branches.

At present, trigeminal nerve tumor is treated mainly by surgical resection to ensure survival. Ideal surgical protocols should be minimally invasive and easily acceptable, being able to relive pain rapidly and entirely without giving rise to complications.^12^ The surgical protocol, which targets the superior maxillary nerve by enlarging the sphenopalatine foramen under endoscopic control and by opening the posterior wall of the maxillary sinus until the foramen ovale, provides a constant positioning marker, thus being more visible and minimally invasive. In the meantime, the maxillary sinus mucosa, which is exposed by endoscopy, is well protected.^13^ The stereotactic positioning used herein allowed accurate and successful surgeries by imaging a clear foramen ovale. As a result, key basicranial structures were protected and the lesions were reached via the shortest route, which minimized the injuries to crucial structures and tissues. In other words, the method was analgesic as well as safe.^14^ All the patients in this study managed to complete the treatment and did not have severe complications. 

In general, trigeminal neuralgia results from excessive synthesis of nerve branch that then transmits pain by decreasing the pain threshold, thus inducing severe, paroxysmal pain. Long-term neurogenic inflammation disorders the release of monoamine neurotransmitters by the central nervous system, which produces noxious stimuli even in the presence of slight maxillofacial tactile stimulation and also leads to sharp pain.^15^ Extracted from the broth of streptomyces and prepared by chemical synthesis, cytarabine is a purine nucleoside antiviral agent, together with its metabolites, can block the synthesis of viral DNA by inhibiting DNA polymerase. This drug moderately inhibits pain by effectively suppressing the synthesis and aggregation of cellular DNA but not those of RNA and proteins.^16^ The pain scores of the two groups herein were similar at T0, T1 and T2, but the score of the treatment group at T2 was significantly different from those at T0 and T1 (P < 0.05).

High-dose cytarabine is not always preferential because of the aggravated adverse reactions that restrict therapeutic effects and extend remission.^17^ In contrast, low-dose cytarabine, which is as effective as standard-dose therapy, is more conducive to the recovery of elderly, critically ill patients owing to milder toxic reactions and shorter hospital stay. In this study, the effective rate of the treatment group (96.7%) was significantly higher than that of the control group (83.3%) (P < 0.05).

**Table-I:** Clinical baseline data of two groups

**Group**	**Treatment (n=60)**	**Control (n=60)**	**χ² or t**	**P**
Gender (male/female)	32/28	31/29	0.083	>0.05
Age (years old)	65.99±2.19	66.13±2.67	0.334	>0.05
Body mass index (kg/m^2^)	21.98±3.18	22.03±4.33	0.192	>0.05
Case distribution (V1/V2/V3)	12/38/10	11/39/10	0.078	>0.05
Disease course (year)	5.34±0.45	5.36±0.55	0.112	>0.05

**Table-II:** Therapeutic effects

**Group**	**Case No.**	**Markedly effective**	**Effective**	**Ineffective**	**Overall effective rate**
Treatment	60	50	8	2	96.7%
Control	60	35	15	10	83.3%
χ²					6.312
P					<0.05

**Table-III:** Pain scores at different time points (point, x ± s).

**Group**	**Case number (n)**	**T0**	**T1**	**T2**	**F**	**P**
Treatment	60	8.32±1.25	3.92±1.26	0.98±0.44	19.234	<0.05
Control	60	8.31±1.36	3.96±1.11	1.00±1.02	18.923	<0.05
t		0.078	0.054	0.072		
P		>0.05	>0.05	>0.05		

**Table-IV:** Complications during treatment (n).

**Group**	**Headache**	**Vomiting**	**Vision impairment**	**Nausea**	**Local swelling**	**Total**
Treatment (n=60)	0 (0.0%)	2 (3.3%)	0 (0.0%)	2 (3.3%)	1 (1.7%)	5 (8.3%)
Control (n=60)	3 (5.0%)	4 (6.7%)	2 (3.3%)	3 (5.0%)	3 (5.0%)	15 (25.0%)
χ²	1.099	0.453	0.583	0.187	0.349	3.234
P	>0.05	>0.05	>0.05	>0.05	>0.05	<0.05

**Table-V:** Quality of life during follow-up period (point, x ± s).

**Group**	**Case number (n)**	**Appetite**	**Sleep**	**Daily living**	**Social life**	**Interest in life**
Treatment	60	5.89±2.34	4.29±1.89	6.34±1.11	6.18±1.76	6.83±1.78
Control	60	2.98±1.27	2.87±1.11	4.98±0.98	5.99±0.76	6.78±1.23
t		7.509	5.498	3.981	0.432	0.132
P		<0.05	<0.05	<0.05	>0.05	>0.05

Although this surgical method is easily operable, a series of complications (e.g. optic nerve injury and local hematoma) may occur because of complex anatomical structures around the foramen ovale. Administration of high-dose drug is bound to increase the risks of headache, nausea and vomiting, so the postoperative quality of life is affected.^18^ During treatment, the treatment group was significantly less prone to complications such as headache, vomiting, vision impairment, nausea and local swelling than the control group (P < 0.05). During three months of follow-up, the appetite, sleep and daily living scores significantly exceeded those of the control group (P < 0.05). Meanwhile, low-dose cytarabine can induce the apoptosis of malignant clone cells, inhibit their proliferation, regulate intercellular adhesion molecules, and eventually facilitate the recovery of patients with tumor.

In summary, stereotactic image-guided surgery successfully treated trigeminal nerve tumor, with its effect boosted by low-dose cytarabine that improved postoperative outcomes and quality of life by significantly decreasing complications.

## Authors Contribution:

**XSL** **&** **BZJ** conceived, designed and did statistical analysis & editing of manuscript.

**XSL, WLY, FZS, GJG & JWM** did data collection and manuscript writing.

**BZJ** did review and final approval of manuscript.

**BZJ** takes the responsibility and is accountable for all aspects of the work in ensuring that questions related to the accuracy or integrity of any part of the work are appropriately investigated and resolved.

## Notes

***Source of funding:*** None.
